# Safety, efficacy and acceptability of praziquantel in the treatment of *Schistosoma haematobium* in pre-school children of Kwale County, Kenya

**DOI:** 10.1371/journal.pntd.0006852

**Published:** 2018-10-17

**Authors:** Bridget W. Kimani, Amos K. Mbugua, Jimmy H. Kihara, Murima Ng’ang’a, Doris W. Njomo

**Affiliations:** 1 Center for Microbiology Research (CMR), Kenya Medical Research Institute (KEMRI), Nairobi, Kenya; 2 Institute of Tropical Medicine and Infectious Diseases, Jomo Kenyatta University of Agriculture and Technology (JKUAT), Nairobi, Kenya; 3 Division of Vector Borne Diseases & Neglected Tropical Diseases (DVBD-NTD), Ministry of Public Health and Sanitation, Nairobi, Kenya; 4 Eastern and Southern Africa Center of International Parasite Control (ESACIPAC), Kenya Medical Research Institute (KEMRI), Nairobi, Kenya; London School of Hygiene and Tropical Medicine, UNITED KINGDOM

## Abstract

**Background:**

The recommended strategy for control of schistosomiasis is preventive chemotherapy with praziquantel (PZQ). Pre-school children (PSC) are excluded from population treatment programs. In high endemic areas, these children are also at risk, and require treatment with PZQ. The Government of Kenya initiated the National School-Based Deworming Programme (NSBDP) where PSC in Early Childhood Development Education (ECDE) Centers are only eligible for treatment with albendazole (ABZ) but not with PZQ.

**Methodology/Principal findings:**

400 PSC were enrolled, from 10 randomly selected ECDE Centers in Kwale County, Kenya where children were treated with crushed PZQ tablets mixed with orange juice, at a single dose of 40 mg/kg. Adverse events were assessed 24 hours post-treatment through questionnaires administered to the parents or guardians. Acceptability was determined by observing if the child spat and/ or vomited all or part of the PZQ dose immediately after treatment. Efficacy was assessed by examining urine samples for *Schistosoma haematobium* eggs in the 5 weeks post-treatment follow-up. Children testing negative for *S*. *haematobium* during the follow-up were considered cured. Egg reduction rate (ERR) was calculated as the decrement in the infection intensity (group’s geometric mean egg counts per 10 ml of urine) following treatment expressed as a proportion of the pre-treatment infection intensity. Before treatment, 80 out of the 400 children enrolled in the study tested positive for *S*. *haematobium* (20.0% (95% confidence interval (CI) 16.4–24.2%). Of these, 41 had infections of heavy intensity (51.3%) while the rest (48.7%) were of light intensity. Five weeks post-treatment, 10 children who had heavy intensity infection were diagnosed with *S*. *haematobium* (prevalence: 2.5% (95% CI 1.5–4.9%). Infection intensities decreased significantly from 45.9 (95% CI: 31.0–68.0) eggs/ 10 ml urine to1.4 (95% CI: 1.1–1.7) eggs/ 10 ml urine during pre-and post-treatment respectively. The ERR was 96.9%. There were no severe adverse events during follow up 24 hours post treatment. Treatment tolerability among the 400 children was high as none of the children spat and/ or vomited as observed in this study.

**Conclusion/Significance:**

The study revealed that crushed PZQ is safe and effective in the treatment of urogenital schistosomiasis in this age group. It is therefore recommended that PZQ should be administered to the PSC in Kwale County.

## Author summary

Control of schistosome infections is through treatment of infected people with a single dose of the anti-helminth drug praziquantel (PZQ) which is safe, highly efficacious, and can reverse schistosome-related morbidity particularly in the early stages of disease progression. However pre-school children are normally excluded due to the belief that these children are not sufficiently exposed to infective water to experience high infection rates. This could lead to clinical manifestation of the disease and the lack of safety data on praziquantel in this age group. Due to this we investigated the safety, efficacy and acceptability of praziquantel in Kwale County, Kenya. We examined urine samples from 400 preschool children. They were treated with crushed praziquantel (40mg/kg) mixed with orange juice and the efficacy of the treatment was determined 5 weeks after treatment. Acceptability was determined by whether the child spat and/ or vomited the treatment through the direct observed treatment (DOT).No child spat or vomited during treatment. Safety of the treatment was assessed by interviewing the parents of the treated children for adverse events (e.g., abdominal pain, dizziness, and headache). The treatment was well tolerated and most of the parasites were cleared by praziquantel.

## Introduction

Human schistosomiasis is a major neglected public health problem caused by trematodes of the genus *Schistosoma*. Over 200 million people are infected globally, with 85% of these cases living in Sub-Saharan Africa [1]. In Kenya, nearly 6 million people are infected and an additional 15 million are at high risk of infection particularly in endemic areas [2, 3]. Schistosomiasis (Bilharzia) is classified as one of the neglected tropical diseases (NTDs). These are a group of diseases found predominantly in tropical areas that are associated with poor sanitation and poverty and which have historically received insufficient attention towards their control. The majority of infections in sub-Saharan Africa are caused by *S*. *mansoni* and *S*. *haematobium* which reside in intestinal mesenteric veins and bladder respectively, leading to intestinal and urogenital schistosomiasis. In Kenya, *S*. *haematobium* occurs mainly in areas around the upper and lower Coast region and some parts of the Lake Victoria and Kano plains in Western Kenya [4]. In affected populations, children carry the heaviest burden of infection [5], [6]. Symptoms of urogenital schistosomiasis include haematuria, dysurea, nutritional deficiencies, anemia, growth retardation, decreased physical performance and impaired memory and cognition [7–10, 1].

Control of schistosome infections is through treatment of infected people with a single dose of the anti-helminth drug praziquantel (PZQ) which is safe, highly efficacious, cheap (costing less than US$0.50/ dose) and can reverse schistosome-related morbidity particularly in the early stages of disease progression [11].

Studies point to a growing body of evidence that in many endemic communities, schistosomiasis infection–contrary to previous beliefs–starts in early childhood. The presence of infection, points to the fact that infants and pre-school aged children are also at risk of infection like their older school-aged counterparts. The growing concern here is that infection in infants and pre-school children (PSC) may persist until the child starts school if left untreated. In preventive chemotherapy control programmes infants and PSC are not eligible for treatment until school-age [12–14].

Failure to reach a majority of the 2–6 year olds in Early Childhood Education (ECDE) Centers could result in higher prevalence of schistosomiasis and its negative health effects such as malnutrition and poor cognitive performance. In turn, these effects retard the child’s growth and development [15]^.^

Treatment of children is also likely to be more successful in averting the development of subsequent, more serious disease sequelae because earlier stages of infection-induced pathology may be reversible if treated promptly [16]^.^World Health Organization (WHO) recommends that young children living in endemic areas be considered for treatment with PZQ during child health campaigns at the standard dose of 40mg/kg [12]^.^

Current schistosome control programmes advocated by the World Health Assembly in 2001 through resolution 54.19 recommend regular de-worming of school age children at risk of infection with anti-helminthes [17]. However, these programs exclude pre-school age children due to the perception that these children are not sufficiently exposed to infective water to experience high infection rates [18]. One of the concerns associated with treatment of preschoolers for schistosomiasis is that they are believed to be at risk of choking on whole tablets. The other one is that there is limited formal data with respect to prescribing information by the pharmaceutical companies on toxicity, method of administration, adverse effects and pharmacokinetics in this age group [19].This may result in clinical disease that is not managed and the lack of safety data on PZQ in this age group [11].

Previous studies have shown that, there have not been severe adverse reactions to PZQ treatment when given to young children due to the excellent safety and tolerability [17]. For administration of PZQ to children under 5 years, it is possible to break the tablet into small pieces or crush them in flavored syrup which would make the tablet palatable and acceptable [17].

The goal of this study was to assess the acceptability, adverse events and efficacy of treating pre-school children with praziquantel for *S*. *haematobium* infection in selected Early Childhood Development Education Centers of Kwale County, Kenya.

## Methods

### Ethical statement

Permission to conduct the current study, including review and approval was obtained from the Scientific Steering and Ethical Review Committees, Kenya Medical Research Institute (KEMRI) SSC No.2958. The county education office, county health office, local leaders, teachers, children and parents/guardians were informed about the study in the area. Written informed consent was obtained from a parent or guardian, for every child in the study. In addition, assent was obtained from the children.

### Study site

This study was conducted in Kwale County, which is situated in the Coast Region of Kenya. It has 4 Constituencies: Msambweni, Lunga-Lunga, Matuga and Kinango. The total population stands at 649,931, of which 36,197 are PSC [20]^.^ Kwale County is mainly an inland county, but it has a coastline south of Mombasa. The area is hot and humid year round with annual mean temperature range of 22°C—34°C, average relative humidity range of 70% - 80%, and annual rainfall range of 900–1500 mm. Altitude ranges from 0 to 462 meters above sea level. The majority of the population 81.9% live in the rural areas with poor road and transport network.Poverty which stands at 71% and lack of sanitation in this area contributes a lot to the high prevalence of soil-transmitted helminthes especially in infants and pre-school children. A large proportion of the population in the study area has no access to safe water and adequate sanitation [21–22]

The current study was conducted in Matuga and Lunga-Lunga constituencies.

### Study design and population

Under the Kenya National School Based Deworming Programme (NSBDP), children in primary schools in the Coast region of Kenya were the first to receive treatment with albendazole and praziquantel. This was after results from a baseline survey in 2011 showed that the prevalence of soil transmitted helminthes and schistosomiasis was high [23].

This sub-study was embedded in a larger study Evaluating Different Drug Delivery Approaches for Treatment of Soil-transmitted Helminthiasis and Schistosomiasis Infections in the NSBDP among Children Attending ECDE Centers in Coast Province, Kenya. SC No. 2547. In the above study, 28 ECDE Centers were targeted for treatment with PZQ.

In the present study, 10 schools were randomly selected from these 28 ECDE Centers. All the children ≤ 6 years of age were enrolled in this study. The study sample was 400 PSC.

This study was a longitudinal, pre and post-test design. Detection of Schistosoma infections was conducted before and after treatment with crushed PZQ mixed with orange juice. The acceptability and safety of PZQ was also assessed. The experimental design entailed laboratory examination of urine samples from the children, where efficacy of the crushed praziquantel mixed with orange juice was determined, by assessing the prevalence and intensity of the *Schistosoma haematobium* eggs pre and post treatment. The descriptive explanatory strategy assessed the acceptability of the crushed praziquantel mixed with orange juice. It also assessed any adverse events after treatment through researcher administered questionnaires to the parents/guardians of the ECDE children 24 hours after treatment. Acceptability was determined by observing if the child spat and/ or vomited all or part of the PZQ dose immediately after treatment. Any adverse events experienced by the children one hour post treatment were observed and recorded by the teachers of the ECDE children and community health extension workers (CHEWs) who took part in the treatment of the children.

### Inclusion and exclusion criteria

Eligibility for inclusion into this study included: 1) aged ≤ 6 years old at recruitment; 2) enrolled in ECDE centers that were targeted for treatment with Praziquantel; 3) production of a urine sample; 4) Parental/guardian consent to participate in the study.

Participants who had existing medical conditions were excluded from the study. These criteria were based on the World Health Organization (WHO) Manual of Preventive Chemotherapy [17]

### Parasitological diagnosis of the infection

Urine samples were collected from all the 400 enrolled children in clean labeled wide mouthed urine containers with lids, between 10 a.m. and 2 p.m. Visible hematuria was recorded upon urine collection. The labeled properly capped containers containing the urine samples were transported with a cool box to the KEMRI Center for Microbiology, Kwale Laboratory for examination.

The urine was thoroughly mixed and a duplicate 10 ml aliquot of urine filtered through 15-mm polycarbonate filters (Nuclear pore R; Costar Europe Ltd., Badhoevedorp, the Netherlands).The filter paper was then placed on a labeled slide and a drop of Lugol’s solution added. The slides were then examined under a microscope within 6 hours and the mean counts of the two filters recorded and expressed as eggs per 10ml urine [17]. The intensity of infection was categorized according to the WHO classification as negative for no detectable eggs; light for 1–49 eggs/10 ml urine; or heavy for > 50 eggs/10 ml urine.

5 weeks post treatment, urine samples were collected from the children who had tested positive for ova of *S*. *haematobium*. This was to assess cure and egg reduction rates [17]. In the present study, a child was considered to have been cured if no *S*. *haematobium* eggs were detected microscopically in urine samples collected 5 weeks post-treatment.

The egg reduction rate was calculated as the decrease in geometric mean intensities of *S*. *haematobium* eggs divided by pre-treatment geometric mean intensity multiplied by a factor of 100.

### Treatment and adverse events

400 PSC were enrolled, tested and treated for *S*. *haematobium* pretreatment. Praziquantel tablets (Prazitel, Cosmos Ltd) were used for treatment in this study. Each child was weighed using a calibrated weighing scale and a single dose of 40mg/kg PZQ administered. Before administration, the PZQ tablets after splitting were crushed with a mortar and pestle and the powder mixed with fruit juice to decrease the bitter taste. This was done during the health break after the children had eaten. Drug administration was supervised using the modified Direct Observation Therapy (DOT). One hour post-treatment observations for any adverse events were made and recorded by the 10 ECDE teachers and CHEWs who took part in the deworming exercise.

Parents or guardians of the treated children were also interviewed using structured questionnaires 24 hours post-treatment for episodes of treatment-related adverse events. The study clinician evaluated the following adverse events abdominal pain, dizziness, nausea, headache, vomiting, drowsiness, itching, as likely or unlikely associated with study drug. Other symptoms reported by parents or guardians were also recorded.

### Treatment efficacy evaluation

Five weeks after praziquantel administration, urine samples from the children who tested positive for *S*. *haematobium* were collected again, using the same procedures. The efficacy of praziquantel was assessed five weeks post treatment using the same diagnostic criteria as baseline. This was determined by means of cure rate (CR, percentage of children positive at the pretreatment cross-sectional survey who became egg-negative 5 weeks after treatment, as assessed by urine filtration for *S*. *haematobium*) and egg reduction rate (ERR, reduction in the group’s geometric mean *S*. *haematobium* egg count in 10 ml of urine comparing the before and after treatment situation) [24].

### Treatment acceptability evaluation

In this study treatment acceptability was defined as the number of children spitting and/or vomiting all or part of the PZQ dose, immediately after treatment and it was assessed by DOT. This was the first time that these children were being treated in school with praziquantel.

### Statistical analysis

Data were double entered in Microsoft Excel spreadsheet. Statistical analyses were done with Statistical Package for Social Sciences (SPSS version 17). PSC who had at least one urine sample subjected to a filtration method for *S*. *haematobium* diagnosis before and after treatment were included in the final analysis. Continuous data (e.g., schistosome egg counts) are presented as geometric mean. Infection intensities were stratified according to the cut-offs defined by the WHO [25].

## Results

### Demographics of enrolled preschool children

The present study enrolled a total of 400 children of preschool age (≤ 72 months). The mean age of the children was 4.8±1.1 years. Those aged three years or less constituting 11.3%, 4 years (26.3%), 5 years (27.5%) and 6 years (35%) of the study sample. Only one child was aged two years. Boys constituted 51.2% whereas girls were 48.8% of the enrolled children.

### Efficacy of praziquantel treatment

#### Parasitological cure rates

The overall pretreatment and post treatment prevalence of *S*. *haematobium* infections was 20.0% (16.4% - 24.2% 95% confidence interval (CI) and 2.8% (1.5% - 4.9% 95% CI) respectively. The parasitological cure rate of praziquantel treatment was thus 86.2% (95% CI 77.0% - 92.1%). The cure rates of praziquantel treatment did not differ with the age or sex of the PSC as shown in Table 1.

**Table 1:** Parasitological cure rate of praziquantel treatment.

Variable	Pretreatment infections		Post-treatment infections		Cure rate		P-value
	No.	% (95%CI)	No.	% (95%CI)	No.	% (95%CI)	
Overall (n = 400)	80	20.0 (16.4–24.2)	10	2.5(1.5–4.9)	70	87.5 (77.0–92.1)	
**Sex**							
Male (n = 205)	47	22.9 (17.7–29.2)	8	3.9 (2.3–8.1)	39	82.9 (67.5–89.6)	0.087
Female (n = 195)	33	16.9 (12.3–22.8)	2	1.0 (0.2–3.7)	31	93.9 (80.4–98.3)	
**Age group**							
≤ 4 years (n = 150)	14	9.3(5.6–15.1)	0	0.0 (0.0–2.5)	14	100.0 (78.5–100.0)	0.102
5 years (n = 110)	19	17.3 (11.4–25.4)	2	1.8 (0.5–6.4)	17	89.5 (68.6–97.1)	0.395
6 years (n = 140)	47	33.6 (26.3–41.7)	8	5.7 (3.4–11.8)	39	83 (67.5–89.6)	REF

#### Effect of praziquantel treatment on infection intensities

Before treatment was conducted, 41 of the 80 children (48.8%) who were infected with *S*. *haematobium* had heavy intensity of infection (≥50 eggs/10 ml urine), whereas 39 children had light intensity of infection (1–49 eggs/10 ml urine). Visible hematuria was observed in 7 children who had heavy intensity of infection. After treatment with Praziquantel, 10 children who were infected with *S*. *haematobium* were found to have an infection of light intensity (1–49 eggs/10 ml urine). These were the children who had heavy intensity of infection (≥50 eggs/10 ml urine) before treatment.

The overall geometric mean of *S*. *haematobium* eggs in the infected children before treatment was 45.9 (95% CI: 31.0–68.0) eggs/ 10 ml urine. Post-treatment geometrical mean intensity of *S*. *haematobium* eggs was 1.4 (95% CI: 1.1–1.7) eggs/ 10 ml urine. The ERR was 96.9% following treatment. Pre-treatment and post-treatment arithmetic mean intensity of *S*. *haematobium* was 146 and 2.7 respectively. The reduction in the intensities of the infection was significant even when the analysis was stratified by sex and age as shown in Table 2.

**Table 2:** *S*. *haematobium* infection intensities.

Characteristic	N	Geometric mean (95% CI) *		P-value
		Before Treatment	After treatment	
Overall	80	45.9 (31.0–68.0)	1.4 (1.1–1.7)	<0.001
**Sex**				
Male	47	59.2 (36.6–101.5)	1.7 (1.2–2.5)	<0.001
Female	33	32.0 (16.8–61.0)	1.1 (0.9–1.5)	<0.001
**Age group**				
≤ 3 years (n = 45)	5	11.0 (2.5–48.4)	1.0 (0.0–1.0)	0.034
4 years (n = 105)	9	42.1 (13.7–129.2)	1.0 (0.0–1.0)	<0.001
5 years (n = 110)	19	32.4 (13.4–78.1)	1.4 (0.7–2.4)	<0.001
6 years (n = 140)	47	62.6 (38.6–101.5)	1.5 (1.1–2.0)	<0.001

*eggs/ 10 ml urine

### Adverse events

Adverse events were assessed 24 hours post treatment. This was through researcher administered questionnaires to the parents and through observations made one hour post treatment by the ECDE teachers and CHEWs, who took part in the deworming exercise.

330 out of the 400 children recruited in the study were assessed for AEs. One experienced dizziness, one experienced a headache, four had abdominal pain/discomfort, two had nausea and two experienced itching. None of the children vomited. While six respondents took no action when their child experienced an adverse event, one gave food, two gave milk and the other one made the child to rest as shown in Table 3.

**Table 3:** Adverse events through parent’s questionnaires 24 hours post treatment.

Characteristic	Number	%
**Experienced any side effects after deworming (n = 330)**		
Yes	10	3.0
No	320	97.0
**Adverse events (n = 10)**		
Vomiting	0	0
Abdominal Pain/discomfort	4	40
Headaches	1	10
Nausea	2	20
Dizziness	1	10
Itching	2	20
**Action taken (n = 10)**		
Given food	1	10
Given milk	2	20
None	6	60
Rested	1	10

### Treatment acceptability evaluation

None of the 400 (100%) PSC spat and/ or vomited during treatment. This was assessed by DOT, by ECDE teachers and CHEWs present during deworming.

## Discussion

Results of the current study showed that praziquantel achieved high cure rates of 86.2% against *S*. *haematobium* infections 5 weeks after treatment. This is in agreement with results of recent Cochrane systematic review which showed that treatment with the standard dose of praziquantel (40 mg/kg) generally results in cure rates of 80% 1–3 months after treatment [24].

In this study, the results show that 10 children out of the 80, who had tested positive for *S*. *haematobium*, had an infection of light intensity (1–49 eggs/10 ml urine) after treatment. These were the children who had infections of heavy intensity of (≥50 eggs/10 ml urine), before treatment. The design of our study did not allow estimating the proportion of infections after treatment that were due to juvenile stages of the parasite, which are largely insensitive to praziquantel. This is in line with a study in Mali assessing urinary schistosomiasis in preschool aged children showing that, the presence of *S*. *haematobium* eggs five weeks post-treatment could be explained by factors such as high pretreatment worm load that could not be completely cleared by the treatment that remained in the treated children and started producing eggs, and the presence of high numbers of immature worms less sensitive to praziquantel that escaped drug action and matured to egg producing worms during subsequent follow-ups [26]; praziquantel is refractory against immature worms [24]. The effect of treatment in terms of egg reduction rates which was 96.9% was high and supported by evidence of Cochrane systematic review [5].

The results of this study revealed that the prevalence of *S*. *haematobium* in pre-school children from Kwale County was high (20%) compared to that observed among school aged in the same county (24.5%) [27]. The findings are consistent with emerging evidence that the burden of schistosomiasis is high in pre-school children. A similar study in Sudan investigating the safety, efficacy and acceptability of praziquantel in pre-school age children reported a prevalence of 31.1% [28], which is higher than what was found in this study (20%). In Ghana, a study investigating the extent of schistosomiasis in pre-school children and infants found prevalence of 11.2% for *S*. *haematobium*, with the highest egg count detected in a 4-month old infant [28], [29]. In a rural endemic area in Nigeria, prevalence of 58.1% was reported for *S*. *haematobium* in children aged 1–6 years [3]. Similar findings have emerged from Mali where prevalence of *S*. *haematobium* among pre-school children aged 1–4 years was found to be 51.2% [26]. In Uganda nearly 50% of children less than three years of age living along the northern shoreline of Lake Victoria had *S*. *mansoni* infections [13]. A recent study from the shoreline villages of Lakes Albert and Victoria in Uganda found even higher prevalence of *S*. *mansoni* (62.3%) in pre-school children [13]. In Sudan, an earlier study found high prevalence of schistosome infection (40%) among pre-school children in the Gezira Irrigation Scheme [30]. The common feature associated with infection in these children from the various settings would be likely due to the fact that the children and their caregivers (parents or guardians) share the common risk factor of proximity to large water bodies known to harbor infectious cerceria. Schistosomiasis in infants and pre-school age children is of concern for at least two reasons. First, this younger age-group plays a hitherto unrealized role in maintaining local disease transmission; even though these infected children may be excreting fewer eggs, it is their regular water contact that leads to contamination of water. Moreover, rinsing and washing children's soiled clothes in environmental water bodies also contributes towards more cryptic contamination and disease transmission [18]. Thus this age-group will play an increasingly important role in environmental transmission likely to frustrate the attempts made by preventive chemotherapy campaigns striving towards more general reductions in environmental transmission [31]. Second, such regular water contact is also likely to result in frequent (re)infection episodes, which lead to a progressive increase of individual worm burden. It is therefore likely that untreated infections acquired in early childhood contribute to worsening the longer-term clinical picture of disease in the individual. Lack of safe water supplies, inadequate sanitation, insufficient access to health care and prohibitive treatment costs all contribute to disease transmission and high morbidities, especially in infection with schistosomiasis. *S*. *haematobium* infection that is predominant in Coastal region of Kenya is found to cluster in a subset of school age children with suggestions of synergistic effects on anemia, cognitive performance and stunting[32]. Chronic anaemia during childhood is associated with impairment in physical growth, cognition, and school performance [33], whereas severe anemia accounts for up to one half of the deaths in children younger than 5 years of age [34].

In Kenya, during the 2009 treatment, only primary school-age children, (6–14 years) both enrolled and non-enrolled were covered by the National School Based Deworming Programme, leaving out the children in the age bracket of 2–6 years who attend the ECDE Centers or pre-school. This age bracket requires to be treated as they also carry a heavy worm burden and pose a risk of re-infecting the treated school-age children while interacting and playing at the community level. In Kenya, the population of children enrolled in ECDE Centers is 2.2 million [35].A high percentage of infected children means that the environment becomes more heavily contaminated–which in turn increases the risk of infection for the whole community. By reducing the number of worms in children, everyone benefits [25].

Given the difficulties of younger children swallowing large PZQ tablets and an associated risk of choking, medications were administered in crushed tablet form and mixed with orange-juice as previously piloted [17].Previous studies have shown that the fruit flavor helped to mask the bitter taste of PZQ [36]. In our study none of the children spat and/or vomited during treatment.

Previous studies have shown that there have not been adverse reactions to PZQ treatment when given to young children due to the excellent safety and tolerability [37]. In this study the adverse events experienced by the children were not severe and included nausea, dizziness, vomiting, abdominal pain/discomfort, itching, and headache which were largely self-limited. This is in line with other studies involving pre-school children, where minor and transient side-effects 24 hours after treatment were reported in Uganda [30], and in Mali [26] in accordance with established evidence that praziquantel is associated with minor and transient adverse events [26, 36] In a study in Sudan there were no drug-related adverse events experienced after treatment with praziquantel [28]. In studies carried out in Uganda and Mali, the adverse events experienced were minor and transient 24 hours after treatment whereas in Sudan, no adverse events were reported.

## Conclusion

In conclusion, the present study showed that crushed praziquantel administered to preschool children at a dose of 40 mg/kg is safe and effective in the treatment of urogenital schistosomiasis. The pre-school children experienced minor side effects which were temporal and most of them required resting under a shade until they subsided. The study also adds to the evidence base that, the prevalence of *S*. *haematobium* in preschool age children is high, and they should be regarded as high risk group in the area, and should be taken into consideration during treatment programs in Kwale County and other endemic regions. This will prevent long-term chronic ill-health or schistosomiasis-related complications later in life.
